# Recognizing patient partner contributions to health research: a mixed methods research protocol

**DOI:** 10.1186/s40900-022-00354-w

**Published:** 2022-06-06

**Authors:** Grace Fox, Dean A. Fergusson, Stuart G. Nicholls, Maureen Smith, Dawn Stacey, Manoj M. Lalu

**Affiliations:** 1 School of Epidemiology and Public Health, University of Ottawa, Ottawa, ON Canada; 2 Clinical Epidemiology Programs, The Ottawa Hospital Research Institute, Ottawa, ON Canada; 3 Departments of Medicine & Surgery, & School of Epidemiology and Public Health, University of Ottawa, Ottawa, ON Canada; 4 Office for Patient Engagement in Research Activities (OPERA), Ottawa Methods Centre, Ottawa Hospital Research Institute, Ottawa, ON Canada; 5 Patient partner, Ottawa, ON Canada; 6 School of Nursing, University of Ottawa, Ottawa, ON Canada; 7 Department of Cellular and Molecular Medicine, University of Ottawa, Ottawa, ON Canada; 8 Department of Anesthesiology and Pain Medicine, University of Ottawa, The Ottawa Hospital, Ottawa, ON Canada

**Keywords:** Patient engagement, Patient partner, Recognition, Financial compensation

## Abstract

**Background:**

The overall aim of this program of research is to assess when/how patient partners are compensated financially for their contributions to health research. The research program consists of three studies to address the following questions: (1) What is the prevalence of reporting patient partner financial compensation? (2) What are researcher and institutional attitudes around patient partner financial compensation? (3) What are the current practices of patient partner financial compensation and what guidance exists to inform these practices?

**Methods:**

In our first project, we will conduct a systematic review to assess the prevalence of reporting patient partner financial compensation and identify current financial compensation practices on an international scale. We will identify a cohort of published studies that have engaged patients as partners through a forward citation search of the Guidance for Reporting the Involvement of Patients and the Public (GRIPP I and II) checklists. We will extract details of financial compensation (type of financial compensation, amount, payment frequency etc.) and reported benefits, challenges, barriers and enablers to financially compensating patient partners. Quantitative data will be analyzed descriptively, and qualitative data will undergo thematic analysis. In our second project, we will conduct a cross-sectional survey of researchers who have engaged patient partners. We will also survey members of their affiliated institutions to gain further understanding of stakeholder experiences and attitudes with patient partner financial compensation. Survey responses will be analyzed by calculating prevalence. In our third project, we will conduct a scoping review to identify all published guidance and policy documents that guide patient partner financial compensation. Overton, the largest available online database of international policy documents, and the grey literature will be systematically searched. Data items will be extracted and presented descriptively. A comprehensive overview of guidance documents will be presented, which will represent a repository of resources that stakeholders can refer to when developing a financial compensation strategy.

**Discussion:**

Our three studies will not only inform and assist patient partners and researchers by informing compensation strategies, but also support the inclusion of diverse perspectives. We will disseminate findings through traditional mediums (publications, conferences) as well as social media, non-technical summaries, and visual abstracts.

## Plain English summary

**Background:**

Partnering with patients, caregivers and members of the public to co-develop and co-conduct research is becoming more accepted and prevalent. However, it is unclear how researchers recognize patient partners for their contributions to research projects. In this publication we outline a series of three studies that will help us better understand when and how patient partners are being financially compensated for their involvement.

**Methods:**

First, we will conduct a review of the literature to identify an international group of researchers that engage patients as research partners. Evaluating these studies will give us an idea about how often patient partners are compensated, and the methods of compensation being used (e.g. offered gifts, cash, etc.). Second, we will survey researchers and institutions who have experience with patient engagement to ask them for more details about their experiences with patient partner compensation (i.e. challenges that they have encountered and their attitudes). Third, we will conduct another review to identify any guidance documents or policies that can help guide researchers through the processes of financially compensating patient partners. We will provide a summary of all of these guidance documents so that researchers, patient partners, and institutions have a one-stop-shop of resources.

**Discussion:**

We hope that our three studies will inform and help researchers and patient partners navigate the processes for compensating patient engagement in research.

## Introduction

Patient engagement in research is defined as the inclusion of patients, defined broadly as any individual with lived experience of a health issue including family members and informal caregivers, as partners in the research process [1–3]. Patient engagement has been observed to enhance research through improved quality, enhanced relevance of research projects and more effective dissemination of study findings [4, 5]. As a result, there is growing evidence of patient engagement in several areas of biomedical research [6–9].

One major challenge to patient engagement is creating an environment where all team members are comfortable discussing elements of the research program and achieving sincere engagement [10]. Tokenistic patient engagement has been highlighted as a major pitfall and patient partners have reported feeling underappreciated. In turn, these experiences can discourage patient partners from continuing to partner with researchers [11]. Compensation (i.e. awarding something to someone in exchange for a service [12]) of patient partner contributions, has been proposed as an important practice to demonstrate how patient partners are valued and acknowledged [10]. Compensation is particularly important when patient partners are allocating their free time to be involved in research. Indeed, “consideration of time and volunteer costs” was identified by a recent study as a key consideration to ensure engagement is sincere [10].

Compensation can take on many forms, including financial (honoraria, salary) and non-financial (co-authorship, services, in-kind exchanges or incentives) constructs [12]. Without financial compensation, patient partners are considered volunteers or individuals who donate their time and energy to benefit others without motivations of financial gain [13]. Volunteerism generally benefits society and can be personally rewarding [13]. In fact, most health charity work is volunteer based and highly influential [14–16]. This may lead researchers to believe that it is unnecessary to compensate patient partners because researchers are providing patient partners with a unique opportunity to inform research development and conduct, which can be perceived as a reward. It has been argued, however, that offering financial compensation may enable members from different socioeconomic populations to be engaged in research and thereby encourage the inclusion of diverse perspectives [16]. This would allow patient partners to decide whether they want to volunteer their time or accept remuneration [16].

For the purposes of this protocol, we will focus on financial compensation, but details of other forms of compensation will be acknowledged wherever possible. While terminology in the domain of patient partner compensation is not standardized, we propose the following definition to describe financial compensation: financial compensation extends beyond the partner’s reimbursement for out-of-pocket expenses and includes offering something of monetary value in exchange for their engagement. Further definitions and examples of compensation can be found in Box 1.

**Box 1:** Terminology used to describe methods of patient partner compensation [12, 17, 18]

Term	Definition
Reimbursement	Reimbursement of out-of-pocket expenses from engagement that are necessary to enable an individual to be engaged as a Patient Partner (travel, accommodations, parking, meals, child-care support or personal healthcare devices such as supplemental oxygen for a plane trip) Reimbursement is not a form of recognition/appreciation/compensation because patient partners should not pay out-of-pocket to be engaged in research
Recognition (non-financial compensation)	Offering gifts, tokens of appreciation or services in exchange for patient partnership on a research project. For example, co-authorship on manuscripts or research material, facilitating patient partner attendance at a conference, education, or gifts (token of appreciation e.g. flowers, care package, gift card)
Compensation (financial compensation)	Financial compensation extends beyond the partner’s reimbursement for out-of-pocket expenses and includes offering something of monetary value in exchange for their engagement. For example, honoraria, cash, or salary (formal payroll) Gifts or gift cards (for grocery stores, restaurants, retail stores, pre-paid visa gift cards etc.) are considered financial compensation when the value is informed by a formal conversion (i.e. 2 h of work at $25 per hour = $50 gift or gift card value or using fair market value) or patient partners decide that they want to receive payment in the form of gifts or gift cards

Yet when it is deemed appropriate to compensate patient partners, several barriers to financial compensation exist [10, 12, 19, 20]. First, it can be challenging to assign a monetary value to a unique skill set and lived experience in a respectful manner [21]. Second, if patient partner compensation is not organized before research funding is secured, it can be difficult to incorporate in a project budget [12, 21]. This may be particularly challenging for researchers who are interested in engaging patients but have no prior experience. Third, offering compensation may alter engagement expectations around the level of commitment and extent of involvement [21]. In turn, this may apply unnecessary pressure on patient partners to be more involved than they wanted, ultimately threatening patient partner autonomy [21]. Finally, it is important to consider the financial and legal implications of remuneration, since financial compensation may be considered taxable income. Thus, financial compensation can interfere with disability payments or income tax rates [17, 18]. Identifying barriers perceived by stakeholders plays a pivotal role in developing mitigation strategies to overcome these barriers.

To date, a few organizations have developed publicly available patient partner compensation policies to provide researchers with the tools necessary to appropriately compensate research partners [12, 17–20]. For example, Canada’s Strategies for Patient Oriented Research (SPOR) networks have developed documents with patient partners outlining items to consider and standards for compensating patient partners [18, 22]. Similarly, the Patient-Centered Outcomes Research Institute (PCORI) in the United States has developed a comparable guidance document to support their members [23]. Additionally, work in this area is being undertaken by groups of individuals with patient partner experience. Richards and colleagues have developed documents that detail some of the existing barriers from the perspectives of patient partners [24]. Despite the observed advances in this area, there remains little empirical data with regards to actual practices of patient partner compensation or the perspectives of researchers and institutions regarding compensation. An understanding of current practices for compensating patient partners, as well as the perceptions toward compensation, will allow us to identify practical and philosophical barriers to compensation as well as enablers from the research institution perspective. Furthermore, we will identify available guidance that is used to inform these decisions, which may prove useful for other researchers and research institutions.

Here we present a program of research consisting of three studies to address this knowledge gap. We will:

1. Assess the prevalence of reporting patient partner financial compensation
2. Identify current patient partner compensation practices (financial and non-financial)
3. Determine researcher and institutional member attitudes and beliefs around financially compensating patient partners
4. Identify perceived barriers to patient partner financial compensation from the perspective of researchers and institutional members
5. Identify and summarize policies and guidance documents that address financial compensation of patient partners

## Methods

### Patient engagement strategy

For the three studies, our research team consists of researchers and a patient partner. All team members have informed project development (e.g. reviewing proposals and protocols, identifying sources, research question generation) and will continue to provide feedback on project conduct (e.g. methodology, drafting non-technical summaries of research findings). All team members will meet regularly and virtually over the project period (and more frequent email communication as needed). Details of engagement will be documented in a terms of reference document to ensure that patient partner expectations of involvement are met. Co-authorship and financial compensation will be offered as a method of acknowledgement. Our patient engagement plan was informed by INVOLVE’s 7 Core Principles of Engagement [25] and the CIHR SPOR Patient Engagement framework [26]. We consulted a larger panel of patient partners to get feedback when developing the project proposal and we plan to continue to consult with similar groups as this research program develops. However, we have opted to work closely with one patient partner who has expertise in systematic review methods. The aim of our collaboration is to ensure that the patient perspective is considered throughout the project. In addition, we will collaborate with a larger panel of patient partners to co-develop a dissemination document that is tailored specifically for this population.

### Study 1: systematic review of published patient engagement research

The objective of our systematic review is to assess the prevalence of reporting patient partner compensation and identify current compensation practices amongst a cohort of studies that engaged patients as research partners. We plan to conduct a systematic review. This protocol was written in accordance with the Preferred Reporting Items for Systematic Reviews and Meta-Analysis (PRISMA-P) [27] extension for protocols and is registered on International Prospective Register of Systematic Reviews (PROSPERO CRD42022303226).

#### Search strategy

The citation function in Scopus and Web of Science will be used to conduct a forward citation search of the Guidance for Reporting the Involvement of Patients and the Public checklists (GRIPP I and II) [28, 29]. Since patient engagement is rapidly emerging in all areas of research [6–9], it would not be feasible to assess the prevalence of reporting patient partner compensation among all published evidence of patient engagement. In fact, a systematic review of patient engagement in clinical trials identified 23 examples of patient engagement from 2777 studies [30]; a similar search across all study types in biomedicine would be too large to feasibly screen. Thus, a forward citation search of the GRIPP checklists is a feasible search strategy to identify a cohort of studies that engaged patients as research partners. There will be no date restrictions, but all identified studies will have publication dates after the first iteration of the GRIPP checklist (2011). There will be no language restrictions, but we anticipate the inclusion of English studies only since the GRIPP checklists are only available in English. If studies in languages other than English are identified, we will compile a list and make it available to readers as a supplemental table in the publication.

#### Eligibility criteria

We will include all studies that both referred to the GRIPP checklists and engaged or described engaging patients as partners in health research. In accordance with the Canadian Institutes of Health Research (CIHR) definition, patient engagement refers to collaboration with patients (i.e., an individual with lived experience of a health condition as well as informal caregivers, including family, friends, or members of patient organizations) as partners (provided input, guidance, consultation or at least one element of the research process) [2]. Engagement may include, but is not limited to, priority-setting, governance, question refinement, defining outcomes, methods and study design, statistical plan, as well as interpretation, dissemination and implementation of results [2]. For the purposes of this review, health research is defined as research aiming to improve our understanding of a human condition (i.e. basic science, clinical research, translational research, health policy etc.). We will include studies that engaged members of the public as research partners. All study designs are eligible including qualitative, quantitative, knowledge synthesis, or mixed-methods research.

#### Selection process

All documents identified by the literature search will be exported and stored in DistillerSR (an audit-ready, cloud-based software program that allows for transparent and reproducible work required for an accurate review) (Evidence Partners Incorporated, Ottawa, Canada). Given that the reporting of patient engagement will likely be included within the main text of the manuscript, title and abstract screening will not be conducted and we will proceed directly to full-text screening. Two reviewers will independently screen studies by full text against the eligibility criteria. Reasons for exclusion will be recorded. The screening process will be piloted for the first 20 articles and will continue (i.e. further sets of 20 articles) until consensus is achieved. Conflicts between reviewers will be resolved by a third-party (MML or DAF) if the two reviewers cannot reach consensus.

#### Data collection process

Two reviewers will independently extract data from studies included in the systematic review. Data extraction will be piloted for the first five studies to ensure reviewers are consistent. After piloting, reviewers will continue to extract data from sets of 20 studies and resolve conflicts between each set. Conflicts will be resolved by a senior investigator (ML or DAF) if the two reviewers cannot reach consensus.

#### Data items

Preliminary data extraction items can be found in “Appendix 1”.

#### Data synthesis

Study characteristics will be synthesized and also presented in tables. The prevalence of reporting patient partner financial compensation will be calculated along with 95% confidence intervals. Study characteristics and details of compensation practices will be extracted and presented descriptively.

Qualitative data (reported benefits, challenges, barriers and enablers to patient partner compensation) will be analyzed by two independent reviewers and in accordance with the 6-step approach developed by Braun and Clark [31]. This process includes generating overarching themes from extracted verbatim statements. Themes will then be collapsed if there are insufficient statements or combined with similar themes. Names will be assigned to themes to reflect the overall message of the included statements and the most compelling themes will be presented in a descriptive manner.

#### Analysis of subgroups or subsets

Prevalence of patient partner financial compensation will be compared between funded and non-funded studies to assess whether a relationship between reported funding and financial compensation exists. Similarly, prevalence will be calculated for the different levels of engagement (consult, collaborate, empower) [32] to investigate whether there is a relationship between the level of patient engagement and patient partner financial compensation. Subgroup analyses will be exploratory.

Our systematic review will provide insights into current practices of financial compensation among a cohort of research teams that have actively engaged patients as partners (a research area with little available evidence). This information, along with any reported barriers or enablers to financial compensation, will inform the development and interpretation of our survey. Additionally, our systematic review findings will identify a cohort of researchers and institutions with patient engagement experience, thus providing the opportunity to delve into greater detail and survey important stakeholders about their experiences and attitudes around patient partner financial compensation.

### Study 2: cross-sectional survey of researchers and affiliated institutions

We will conduct tailored cross-sectional surveys of (1) researchers who have engaged patients as partners, and (2) their affiliated institutions. The objectives of our survey are to answer the following questions:

1. How are researchers and institutions **recognizing** patient partners for their contributions to research (e.g. financial and non-financial practices)?
2. What are the **barriers** to financially compensating patient partners from the perspective of researchers and institutional members?
3. What are researcher and institutional member **attitudes** around financially compensating patient partners?

We will register the full survey protocol on Open Science Framework. The survey study will undergo review by the Ottawa Health Science Network Research Ethics Board.

#### Sampling strategy

Irrespective of reporting patient partner compensation, all corresponding authors of the articles included in the systematic review will be invited to participate in the survey (i.e. researchers with experience engaging patients as partners). Corresponding author institutional affiliations will be used to identify institutions that have experience supporting patient engagement in research.

#### Survey question generation

Survey questions will be generated with input from all team members, including patient partners. We will use existing patient engagement literature and studies identified by the systematic review to construct the first draft of questions (e.g. type of financial compensation, potential barriers to financial compensation). All team members will reflect on their experiences with patient partner financial compensation when reviewing questions. Survey questions will undergo several iterations of revision and refinement and all members will approve final survey questions. One team member has experience conducting surveys and has received exceptional response rates (90% +) [33]. Two survey characteristics that may explain such a high response rate are that the survey invitation came from a familiar name and was concise (3–5 questions) [33]. Thus, our survey will be purposefully short (approximately 5 questions).

#### Pilot procedure

We will pilot the survey with international researchers with experience in patient engagement in research to ensure usability and question clarity. Researchers will be asked to fill out the survey based on their experiences with patient partner compensation. They will have the opportunity to provide feedback on the questions and suggest additional response options. Amendments will be made prior to submission for ethics approval.

#### Survey administration 

Survey procedures will be informed by validated methods developed by Dillman [34]. Researchers and institutions will be contacted using their publicly available e-mail address with a link to the online survey hosted by Lime Survey (Lime Survey GmbH, Germany). Participants will be reminded by email at 1-, 3- and 7-weeks after the initial invitation is sent [34].

#### Analytic plan

All survey questions will be close-ended questions (e.g. multiple choice, Likert scale). Participant responses will be analyzed and presented descriptively. Proportions and 95% confidence intervals will be calculated when appropriate.

This cross-sectional survey will provide an in-depth perspective of researcher and institutional member experiences and attitudes around patient partner financial compensation. Survey findings will ultimately identify any perceived barriers to patient partner financial compensation, thus highlighting avenues to overcome these barriers.

### Study 3: scoping review of guidance documents

We will conduct a scoping review to identify and summarize current patient partner compensation policies and guidance documents. The overarching research question is *what guidelines or policies exist to guide patient partner compensation in health research?* For the purposes of this review, we will be using the same definitions outlined above for *patient partner*, *patient engagement* and *financial compensation* (Study 1: systematic review of published patient engagement research and Box 1). We will follow a standard approach to conducting scoping reviews [35]. This protocol is written in accordance with work conducted by Peters and colleagues mapping reporting items from the PRISMA protocol [27] extension to scoping review items [36]. We will register the full scoping review protocol on Open Science Framework.

#### Search strategy

Overton.io (the largest available online database of international policy documents) and the grey literature will be systematically searched. The grey literature search will be conducted in accordance with the Grey Matters tool designed by the Canadian Agency for Drugs and Technologies in Health [37]. Searches will be co-developed with an information specialist.

We will limit the search to studies published during or after 2000 to capture policy documents that reflect current patient partner compensation practices. We will only include policies written in English. However, we will compile a list of policies that were excluded based on language and make this accessible to interested readers.

#### Eligibility criteria

As per scoping review methodology [35], we have prespecified eligibility criteria but will amend criteria as we become familiar with the literature. All documents identified by the database searches will be screened against the following eligibility criteria:

- **Types of evidence sources:** Policy documents or reports that have been developed to guide patient partner compensation.
- **Population:** Not applicable.
- **Concept:** Guidance to compensate patients or members of the public in their role as patient partners in research, consultants, members of steering/advisory committees, holding positions on grant review committees etc.
- **Context:** Guidance documents developed by any national organization will be included.

#### Selection and data extraction process

Screening and data extraction processes will be identical to those outlined above for Study 1: systematic review of published patient engagement research. If multiple versions of the same policy document are identified, the most recent version will be included. Additionally, reviewers will confirm that included policies or guidance documents are the most up to date versions.

#### Data items

Preliminary data extraction items can be found in “Appendix 2”.

#### Data analysis

Data will be presented descriptively in tables. We will review extracted benefits, challenges, barriers and enablers to patient partner financial compensation to identify reoccurring concepts. Two reviewers will review the extracted statements independently before meeting to compare concepts. These concepts will be presented to provide an overview of reported benefits, challenges, barriers and enablers to patient partner financial compensation.

Scoping review findings will provide researchers, patient partners, institutional members and policy makers with a comprehensive overview of international guidance documents and policies that guide patient partner financial compensation. Ultimately, this comprehensive overview will compare and contrast all available documents and act as a one-stop-shop of resources for stakeholders to refer to when developing a patient partner compensation strategy.

## Discussion

Despite the uptake of patient engagement in health research, there exists little empirical evidence on how patient partners are recognized for their contributions [6–9]. Furthering our understanding and expectations of patient partner financial compensation will not only affect individual patient partners and researchers by informing compensation strategies (i.e. stakeholders are made aware of the different methods to recognize patient partners and the benefits of recognition), but it may also encourage diversity by including those with financial or income constraints. Our research program will present the various approaches to patient partner recognition as well as possible explanations for variation by highlighting reported barriers and enablers to compensation practices.

A few limitations of our approach should be noted. First, we do not assess the prevalence of reporting patient partner financial compensation in all published examples of patient engagement in health research. However, given that patient engagement is becoming increasingly popular in all areas of research, it is not feasible to conduct a broad literature search. With that said, our search strategy provides a highly specific alternative; since GRIPP I and II are recognized standards for reporting patient engagement in research, we anticipate that the studies that cite these reporting checklists are more likely to also report on patient partner compensation practices*.* Second, our survey will capture the perspectives of researchers and institutional members while omitting those of patient partners. Despite the importance of the patient perspective, the scope of the present study is to assess researcher and institutional barriers to the provision of compensation. We anticipate that the barriers faced by patient partners will be unlike those experienced by researchers and institutional members. Thus, future work will focus on identifying patient perceived barriers to compensation. Finally, Overton is a relatively new database with little published evidence of utilization for research purposes (https://www.overton.io/). Despite this, Overton is the largest available databases of international policy documents and has been recently validated to support systematic searches [38]. Additionally, we will conduct a grey literature search as this provides a pragmatic approach to identify policies pertaining to patient partner financial compensation. We will supplement findings from the scoping review search with any reported guidance documents identified by the systematic review and survey study. We will also utilize social media to identify any additional documents.

Given the nature of our topic, we will prioritize knowledge translation and dissemination of research findings. This includes ensuring that communications are formatted in ways that are accessible to a broad audience. We will co-develop non-technical summaries and visual abstracts for each project with a patient partner. Additionally, we will create an open-access repository of guidance documents identified by the systematic review, survey and scoping review to provide a one-stop-shop for researchers who are developing a patient partner compensation strategy as well as a document tailored specifically for patient partners outlining a list of items to consider regarding compensation. The repository of guidance documents will be stored on the Office for Patient Engagement in Research Activities (OPERA) website and the Open Science Framework. We will also co-develop, with patient partners, a document for patient partners that contains a list of items to consider with regards to compensation. Dissemination material will be circulated by social media to reach experienced and inexperienced patient partners, researchers, and policy makers. Second, we will leverage relationships with various patient centred institutions to circulate documentation and the repository to their members and networks (e.g., Ontario SPOR SUPPORT Unit, SPOR Networks). We will extend this invitation to other institutions or funding agencies that are interested in our research findings. Lastly, in order to reach an even broader audience, we will publish each project as an individual manuscript in an open access journal and ensure transparent and complete reporting by using appropriate checklists [39–41].

## Data Availability

Not applicable.

## Appendix 1: Systematic review data extraction items

**Table:**

Data item	
1. Corresponding author name, e-mail address, country of residence, and institutional affiliation at time of publication 2. Publication title 3. Year of publication 4. Journal/Source of publication 5. Funding details (e.g. source of funding, whether funding was received specifically to support patient engagement) 6. Type of stakeholder engaged (e.g. patients, caregivers, community member etc.) 7. Number of patient partners engaged 8. Length of engagement (i.e. whether patient partners were engaged once or multiple times throughout the project) 9. Research element where patient partners contributed (e.g. funding, priority-setting, governance, study design, data collection, data analysis, dissemination, ethics approval etc.) [2] 10. Level of patient partner engagement (as defined by INVOLVE [32]) 11. Non-financial compensation offered to patient partners (e.g. co-authorship, gifts, refreshments etc.) 12. Did authors report on offering financial compensation to patient partners (patient partners need not accept)? (Yes or No) 13. Where are details of financial compensation reported in the manuscript? (e.g. methods, results, discussion) 13 a) Type of financial compensation (e.g. honoraria, salary, cash etc.) 13 b) Amount (rate and total) 13 c) Payment frequency (e.g. bi-weekly, one-payment etc.) 13 d) Reported guidelines or policies used to guide financial compensation 14. Stated reason for financially compensating patient partners or stated reason for not financially compensating patient partners 15. Reported benefits and challenges to financially compensating patient partners 16. Reported barriers and enablers to financially compensating patient partners	

## Appendix 2: Scoping review data extraction items

**Table:**

Data item	
1. Author details 2. Year of publication 3. Geographical location 4. Target population (e.g. patients which chronic diseases, children, elderly population, Indigenous peoples etc.) 5. Details of compensation (e.g., type of payment, frequency, amount etc.) 6. Reported benefits or challenges of patient partner compensation 7. Reported barriers or enablers to guidance document implementation 8. Special considerations when compensating patient partners	

## Abbreviations

GRIPP: Guidance for Reporting the Involvement of Patients and the Public
CIHR: Canadian Institutes of Health Research
OSSU: Ontario SPOR SUPPORT Unit
SPOR: Strategies for Patient-Oriented Research
